# The Editor's Letter-Box

**Published:** 1913-03-29

**Authors:** J. Gordon Macqueen


					Having been furnished with copies of the above letters,
the following is Dr. Macqueen's reply :
Sm,?I have read the above letter signed " Mabel Cicely
Wells." It forms interesting reading, but it is easily
answered. I think that in provincial hospitals, the phrase
"Ma's Pets" is well known; and it wars applied to the
writer of the letter by some of her co-workers in the
institution.
By her withdrawal of the words " and in some cases
untrue " she contradicts herself. As the letter first stood
she admits that some of the charges are true?some, accord-
ing to her, untrue?and if her withdrawal of the words
be taken to mean that the charges are true, then why saj
more ? if, on the other hand, by her withdrawal she mea?s
to insinuate that all are untrue, then she has contradicted
herself.
She is the first whom I have heard say that the food *n
that institution was excellent?all othei-s, whom I heard
speak of it, had tho opposite tale. The food which ntf
colleague and myself were served with has already bee11
described, and I do not think that the nurses would faf0
better than the residents.
I was conversing with a pi-evious resident on Saturda}
last,, and he informed me that he would have written a?
article supporting some of the statements: pf Dr. Shepherd
and myself ,only; having, seen ? the announcement in
papers that there was to be an inquiry he had decided
reserve his evidence until then.?I am, Sir, yours fait"
fully, (Signed)
J. Gordox Macqtjeex.

				

